# Molecular mechanisms of memory in imprinting

**DOI:** 10.1016/j.neubiorev.2014.09.013

**Published:** 2015-03

**Authors:** Revaz O. Solomonia, Brian J. McCabe

**Affiliations:** a Institute of Chemical Biology, Ilia State University, 3/5 K Cholokashvili Av, Tbilisi 0162, Georgia; b I. Beritashvili Centre of Experimental Biomedicine, Tbilisi, Georgia; c University of Cambridge, Department of Zoology, Sub-Department of Animal Behaviour, Madingley, Cambridge CB23 8AA, United Kingdom

**Keywords:** Learning, Memory, Behavioural imprinting, IMM

## Abstract

Converging evidence implicates the intermediate and medial mesopallium (IMM) of the domestic chick forebrain in memory for a visual imprinting stimulus. During and after imprinting training, neuronal responsiveness in the IMM to the familiar stimulus exhibits a distinct temporal profile, suggesting several memory phases. We discuss the temporal progression of learning-related biochemical changes in the IMM, relative to the start of this electrophysiological profile. *c-fos* gene expression increases <15 min after training onset, followed by a learning-related increase in Fos expression, in neurons immunopositive for GABA, taurine and parvalbumin (not calbindin). Approximately simultaneously or shortly after, there are increases in phosphorylation level of glutamate (AMPA) receptor subunits and in releasable neurotransmitter pools of GABA and taurine. Later, the mean area of spine synapse post-synaptic densities, N-methyl-d-aspartate receptor number and phosphorylation level of further synaptic proteins are elevated. After ∼15 h, learning-related changes in amounts of several synaptic proteins are observed. The results indicate progression from transient/labile to trophic synaptic modification, culminating in stable recognition memory.

## Highlights

• We discuss learning-related biochemical changes in a chick brain memory system.
• These changes reflect neuronal responsiveness to the imprinting stimulus.
• Early changes occur in *c-fos*, synaptic protein phosphorylation and transmitter pool.
• Intermediate changes involve transmitter pool and glutamate NMDA receptors.
• Late changes (with maximum neuronal responsiveness) involve protein synthesis.

## 1 Introduction

**Fig. 1:**
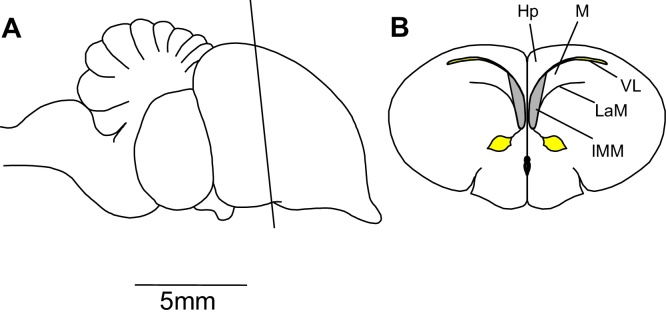
Position of the IMM. A, side view of the chick brain, indicating the plane of section for B. B, coronal section; Hp, hippocampus; IMM, intermediate medial mesopallium; LaM, lamina mesopallialis; M, mesopallium; VL, lateral ventricle.

Over the past few decades, there has been substantial progress in understanding the mechanisms of learning and memory. This progress continues to depend on a combination of complementary experimental approaches on a variety of experimental animals. The domestic chick is one such animal and offers a number of important advantages. First, a region of the chick forebrain has been identified as being of crucial importance for the learning process of filial imprinting, the available evidence indicating that it is a site of storage of information about a visual stimulus to which a chick has become imprinted. This region is the intermediate and medial mesopallium (IMM), formerly known as the intermediate and medial hyperstriatum ventrale (IMHV) (Reiner et al., 2004). The IMM is a bilateral structure adjacent to the lateral wall of the lateral ventricle (Fig. 1) and makes up approximately 3% of the telencephalon by volume. The evidence implicating the IMM as a memory system for imprinting is summarized extensively elsewhere (see e.g. Horn, 1985, Horn, 2004, McCabe, 2013). The IMM is sufficiently localized to be targeted by lesion experiments, to be probed with microelectrodes (see Nicol and Moorman, 2015) and to be subjected to quantitative histological (Bradley et al., 1981, Horn, 1985, McCabe and Horn, 1994, Suge et al., 2010, Suge and McCabe, 2004) and biochemical analysis (Horn, 2004, Solomonia et al., 1997, Solomonia et al., 1998, Solomonia et al., 2003, Solomonia et al., 2005, Solomonia et al., 2008, Solomonia et al., 2011, Solomonia et al., 2013). The IMM also plays an essential role in single-trial passive avoidance learning in the chick (see e.g. Rose, 2000). Here, we focus on imprinting.

In addition to the availability of the IMM as a memory system for study, research into neural mechanisms of imprinting is aided by the fact that behavioural aspects of imprinting and the neurobiology of the avian brain have been extensively documented. It is also an important advantage that the visual experience of the recently hatched chick has been minimal and can be controlled, facilitating the detection and analysis of learning-related changes in the brain. The fact that the chick does not require food or water for the early part of the typically three- to four-day sensitive period for filial imprinting removes a complication inherent in animals that must be fed. Newly-hatched chicks are very active and vocalize extensively, offering abundant behavioural read-out from which learning and memory-related behaviour may be inferred. Imprinting is a powerful and rapid form of learning, associated with processes having the characteristics of recognition memory observed in a range of animals (Bateson, 1990). Features of imprinting memory closely resemble recognition memory in mammals, suggesting that mechanisms revealed in the chick may exist in other vertebrates.

This review will concentrate on biochemical changes occurring in the IMM at various times in the first day after imprinting training. Concomitant electrophysiological changes in the IMM are described and discussed elsewhere (Nicol and Moorman, 2015). We shall attempt to keep these two sets of observations in register and comment on ways in which they might be connected. Where the opportunity arises, we shall attempt to relate neural changes observed in studies of imprinting to changes arising from learning and neural modifications observed in other species.

The accumulated evidence strongly indicates that formation of the recognition memory of imprinting involves a time-dependent chain of biochemical processes in the IMM. Such processes are clearly expressed in the left side of this region. Changes in the right IMM after training, though resembling events in the left in some respects, have a different temporal pattern, as might be expected from the functional lateralization of the IMM (McCabe, 1991, McCabe, 2013, Nicol and Moorman, 2015, Rose, 2000). Research on biochemical learning-related changes has focussed mainly on the first day after training, and this may conveniently be divided into three parts: (1) Early changes (detected up to ∼7 h after the start of training); (2) Intermediate changes (detected ∼7–15 h after the start of training) and (3) Late changes (detected >15 h after the start of training).

All results described here employed an artificial training stimulus of the type that was also used for the electrophysiological experiments mentioned above. That is, chicks were trained by exposing them to either a rotating, internally-illuminated red box or to a rotating, internally illuminated blue cylinder as described, for example, by Bolhuis et al. (2000).

## 2 Early changes (up to ∼7 h after the start of training)

### 2.1 *c-fos*

The expression of the immediate-early gene *c-fos* can be induced in some neurons on stimulation (Herdegen and Leah, 1998, Herrera and Robertson, 1996). Expression of this gene and its product Fos have accordingly been used extensively as neural activity markers.

**Fig. 2:**
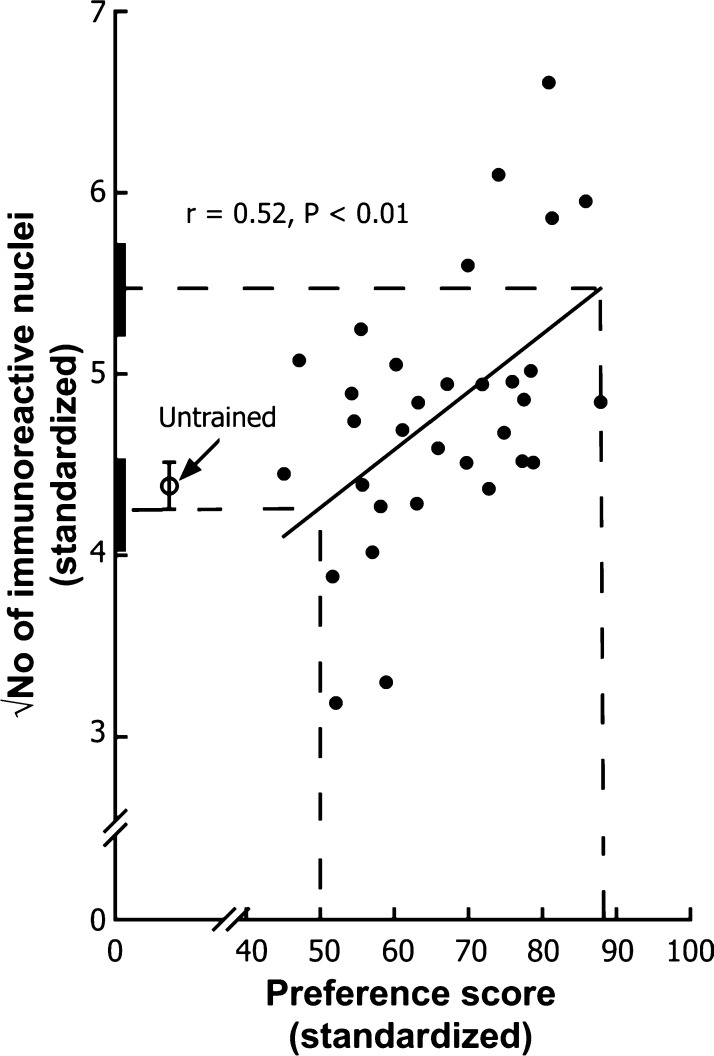
Fos expression in the IMM after imprinting. Number of Fos-positive nuclei per unit area (square root-transformed to normalize the data) are plotted against preference score, a measure of preference for the imprinting stimulus and thus of the strength of imprinting/learning. Nuclei were counted in a standard sampling frame placed over the IMM region in a histological section. Each point represents data from one chick. The least squares regression line has been fitted. The lower horizontal dashed line estimates the value of the ordinate corresponding to the ‘no preference’ score of 50 (characteristic of chicks showing no learning). This estimate was not significantly different from the mean value for untrained chicks, which is represented by the open circle (the error bars represent ±1 SEM, *n* = 16). The upper horizontal dashed line gives the estimated value of the ordinate corresponding to the maximum preference score attained in the experiment (characteristic of strongly imprinted chicks). This estimate was significantly greater than the mean value for untrained chicks. The estimates shown by the horizontal dashed lines are based on interpolation of the regression line. The thick bars on the *Y* axis depict ± one standard error of each estimated value.

Reproduced with permission from McCabe (2013).

Training for 60 min with a visual stimulus accompanied by the maternal call of a hen typically results in strong visual imprinting in some chicks but less strong imprinting in others, permitting correlations to be run between a behavioural measure of the strength of learning and a physiological variable potentially involved in learning or memory. An appropriate measure of learning strength is the preference score, derived from a test in which a chick, previously trained by exposure to an imprinting stimulus, is exposed in turn to this (by now familiar) stimulus and to a novel stimulus; the preference score is the approach activity in the test directed towards the familiar stimulus expressed as a percentage of total approach in the test, directed towards both familiar and novel stimuli. Behavioural protocols of this nature, namely training by exposure to an imprinting stimulus followed by a preference test to measure strength of learning, were employed in the experiments described subsequently in this review. McCabe and Horn (1994) found that preference score measured 10 min after a 1 h training session was positively correlated with the number of IMM cells immunoreactive for Fos, measured 2 h after the start of training. Measurements were also made on untrained chicks, which remained isolated in a dark incubator throughout the experiment. The least-squares straight line describing the regression of Fos expression on preference score was interpolated to a preference score of 50, indicative of no learning. The level of Fos expression corresponding to this no-learning preference score was very similar to, and not significantly different from, the mean level in untrained chicks. Thus, placing chicks in running wheels, allowing them to see and run towards the training stimulus, and vocalize as they did so, did not change Fos expression in the IMM: side-effects of training had no effect on Fos expression in the IMM and it was evidently necessary for learning to occur before a change in Fos expression could be detected in this region. On the other hand, the level of Fos expression corresponding to the highest preference score estimated from the regression was significantly greater than the untrained value (Fig. 2). No lateralization of this effect was observed.

Further information is available from this experimental design. A correlation between preference score and Fos expression does not necessarily imply a causal relationship. It is possible, for example, that chicks constitutively expressing high levels of Fos in the IMM can learn more than chicks constitutively expressing low levels of Fos. A positive correlation between preference score and Fos expression would then be expected without the learning process affecting Fos expression at all. McCabe and Horn (1994) called this scenario the ‘predispositions’ hypothesis. The alternative hypothesis is that learning causes an increase in Fos expression. One may distinguish between these two hypotheses by dividing the trained chicks into two sub-groups of equal size – good learners and poor learners – on the basis of their preference scores. One may then compare the residual variance about each sub-group mean with the variance of the untrained chicks. If the predisposition hypothesis were correct (no effect of learning on Fos expression), the variances of both untrained and trained chicks would be estimates of the same population variance. The mean levels of Fos expression in the good learners and poor learners of the trained group differed significantly from each other. The predispositions hypothesis predicts that the respective residual variances about the means of the good and poor learners should be significantly *lower* than the variance of the untrained chicks, since a significant difference between good and poor learners implies a reduction in the residual variance about their respective means (cf Horn and Johnson, 1989). In fact (McCabe and Horn, 1994), these residual variances were each *greater* than the variance of the untrained chicks. This is the opposite of what is predicted by the predisposition hypothesis (McCabe, 2013, McCabe and Horn, 1994). The predispositions hypothesis was therefore rejected in favour of the conclusion that learning causes an increase in Fos expression in the IMM.

When testing the predispositions hypothesis, an alternative to dividing chicks into good and poor learners is to take information from the regression line relating Fos expression to preference score. If there is a significant correlation between these two measures, the residual variance about the regression should be significantly smaller than the variance before fitting the model. Whereas it is also possible for such a reduction in variance to occur when Fos expression has been influenced by learning (for example, in the extreme case there might be a perfect correlation between learning and Fos expression), a reduction in variance is a necessary consequence of a predisposition. If such a reduction does not occur, the predispositions hypothesis has no support.

Training for 1 h can thus produce strong imprinting in some chicks and there is good evidence that the resulting increase in Fos expression in the IMM reflects memory formation. Fos protein is a component of the AP-1 transcription factor (Herdegen and Leah, 1998), but it is not known which late-response genes are controlled by the change in Fos expression in this particular instance. The results do, however, indicate which neurons are active in the IMM in association with memory formation. Subsequent double-labelling experiments (Ambalavanar et al., 1999) showed that the learning-related change in Fos expression in the IMM occurs in neurons that are immunopositive for γ-aminobutyric acid (GABA), implying that they are inhibitory, and occurred in the sub-set of GABA-positive neurons which are immunopositive for parvalbumin and not calbindin. The cells were also immunopositive for the amino acid taurine and for protein kinase C-γ (Ambalavanar et al., 1993, van der Zee et al., 1995). A population of inhibitory neurons involved in early stages of memory formation was thus identified.

Expression of *c-fos* mRNA precedes Fos expression in the IMM by approximately 1 h (Suge et al., 2010). In the study by McCabe and Horn (1994), Fos expression was measured 120 ± 10 min after the start of training, a preference test lasting 15 min having been administered 70 min after the start of training. The learning-related increase of Fos expression therefore evidently reflected events occurring early in memory formation. The timing of these early events was investigated by Suge and McCabe (2004), who trained chicks for periods of between 15 and 60 min. The time courses of Fos expression in the IMM for these training times strongly resembled each other, and expression in both cases was significantly positively correlated with preference score. It was therefore concluded that the IMM is the site of learning-related events within the first 15 min of imprinting training. Expression of *c-fos* mRNA in the IMM during training was measured by Suge et al. (2010), who found a peak increase in *c-fos* transcription 15 min after the start of training, implying that transcription started even earlier than this peak. These results do not exclude the possibility of *c-fos* expression increasing after this early stage, but in the IMM, *c-fos* expression had returned to baseline 90 min after the start of training and Fos expression had returned to baseline 60 min after that (Ambalavanar et al., 1999, Suge and McCabe, 2004).

At this early stage of imprinting, the effect of learning on Fos expression did not differ significantly between the left and right sides of the IMM. In contrast to the asymmetries found in the IMM somewhat later (see below), it is evident that neuronal activity changes in a learning-related manner in both the left and right IMM during the first hour of training.

The expression of the *c-fos* gene during and after imprinting has been studied in vivo by Yamaguchi et al. (2010), visualizing expression of the gene through coupling with luciferase activity (Yamaguchi et al., 2007). An increase in gene expression as a result of training with a visual stimulus was found, together with a significant positive correlation between gene expression, and both a measure of preference after training (i.e. strength of imprinting) and approach during training. When partial correlation (see e.g. Snedecor and Cochran, 1989) is used to adjust for the association of both of these measures with approach activity during training, a positive trend remains, although this does not reach statistical significance, possibly owing to sample size.

The criteria employed to determine whether the changes in Fos expression are related to learning have also been applied to many biochemical changes discussed in the remainder of this article: the term ‘learning-related’ has been employed when these criteria are satisfied (see McCabe, 2013 for further discussion).

### 2.2 αCalcium-calmodulin dependent protein kinase II (αCaMKII)

αCaMKII is abundant in neurons and has been assigned critical roles in the form of synaptic plasticity known as long-term potentiation (Lisman et al., 2012, Lisman, 1985). When the kinase has been activated by exposure to Ca^2+^/calmodulin, autophosphorylation can occur at Thr286 and in consequence the kinase remains active after Ca^2+^ is withdrawn (Hudmon and Schulman, 2002, Miller and Kennedy, 1986). This property of the kinase has prompted the proposal that it plays an essential part in the prolonged modification of synaptic efficacy. It is proposed to do this by phosphorylating glutamate receptors of the α-amino-3-hydroxy-5-methyl-4-isoxazoleproprionic acid (AMPA) subtype, thereby increasing their channel conductances, and by phosphorylating stargazin protein, resulting in an increase in the number of sub-synaptic AMPA receptors. Association of αCaMKII with glutamatergic N-methyl-d-aspartate (NMDA) receptors at the sub-synaptic membrane appears to be an important part of this process. αCaMKII has been proposed to be a molecular switch, persisting in an activated state after Ca^2+^/calmodulin has been withdrawn, and thus encoding synaptic modification pending further, more long-term synaptic changes. It has been suggested that this synaptic modification plays a critical role in memory formation (see Lisman et al., 2012 for review).

We tested this hypothesis by measuring the total amount of αCaMKII (tCaMKII) and the amount of Thr286-autophosphorylated αCaMKII (apCAMKII) in the IMM and in a control region, the posterior pole of the nidopallium (PPN), in which learning-related changes have not been found in experiments where such changes occurred in the IMM (Solomonia et al., 1997, Solomonia et al., 1998, Solomonia et al., 2003, Solomonia et al., 2005, Solomonia et al., 2008, Solomonia et al., 2011, Solomonia et al., 2013). These measurements were made either 2 h or 25 h after the start of imprinting training. At 2 h there was a learning-related increase in the ratio apCAMKII/tCaMKII in the IMM: learning was followed by an increase in the proportion of enzyme molecules that had undergone autophosporylation. This increase was regionally specific (it was observed in the IMM but not in the PPN) and time-dependent: at 25 h no changes were detected in either of the two brain regions studied (Solomonia et al., 2005). Thus, 2 h after the start of training, the level of αCaMKII autophosphorylation has increased selectively in the IMM, consistent with previously described mechanisms of use-dependent synaptic modification. These results do not exclude the possibility that the autophosphorylation of αCaMKII is involved in long-term memory but indicate that, for imprinting, the processes contributing to memory formation change over time, the earlier processes relying on the phosphorylation at Thr268 in a way that later processes do not.

Mice with a mutation preventing autophosphorylation of αCaMKII show impairment of passive avoidance learning and fear conditioning but are nonetheless able to acquire these tasks with repeated training, leading to the suggestion that αCaMKII autophosphorylation is necessary for learning under certain circumstances, such as those requiring only one or a few training trials, but can be circumvented by other pathways when training is prolonged or particularly effective (Irvine et al., 2006). Our results are consistent with this interpretation.

#### 2.2.1 Processes downstream of activated αCaMKII

The learning-related autophosphorylation of αCaMKII 2 h after the start of training raises the question of which signalling pathways are activated by the activity of this enzyme during memory formation. An important target of αCaMKII is the GluA1 subunit of the AMPA receptor. Activated αCaMKII directly phosphorylates Ser831 in the C terminus of GluA1, promotes its delivery into the postsynaptic membrane and increases the channel conductance of AMPA receptors (Barria et al., 1997, Derkach, 2003, Kristensen et al., 2001, Lee et al., 2000, Lisman et al., 2002). Phosphorylation of GluA1 at Ser831 by αCaMKII is involved in the induction and maintenance of long-term potentiation (Barria et al., 1997, Lee et al., 2000) and has been implicated in some types of memory (Banks et al., 2012, Cui et al., 2011, Hu et al., 2007, Takeuchi et al., 2014).

We inquired whether the learning-related increase in the autophosphorylation of αCaMKII is followed by changes in the Ser831 phosphorylation of GluA1. Our original αCaMKII studies were conducted with a 1-h training protocol and biochemical measurements were made 2 h and 25 h after the start of training (Solomonia et al., 2005). Since a learning-related increase in Fos-like immunoreactivity is achieved also with shorter periods of training (Suge and McCabe, 2004), in one series of experiments chicks were trained for 30 min. The results from chicks trained for 1 h and from those trained for 30 min were statistically homogeneous and the data from both series of experiments were therefore pooled. It was found that, in the left IMM 2–2.5 h after the start of training, the amount of Ser831-phosphorylated GluA1 expressed as a fraction of the amount of total GluA1 (pGluA1/tGluA1 ratio) increased in a learning-related way. A similar (albeit non-significant) trend was found in the right IMM and there was no evidence of any learning-related changes in either the left or the right PPN. No significant changes were observed in the total amount of GluA1 in any brain region studied (Solomonia et al., 2013). Nor were there such changes in the pGluA1/tGluA1 ratio, or in the amounts of pGluA1 or tGluA1, at 25 h (Solomonia et al., 2013). Phosphorylation of the Ser831 on the GluA1 subunit has been found approximately to double the conductance of the AMPA channel (Kristensen et al., 2001). The results indicate that a time- and region-dependent, learning-related increase in GluA1 phosphorylation occurs early in recognition memory formation, possibly leading to increased channel conductance of AMPA receptors (Solomonia et al., 2013).

**Fig. 3:**
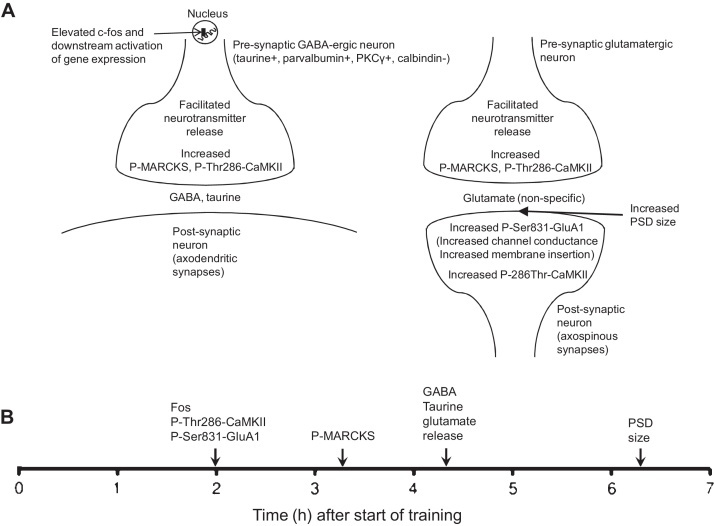
(A) Hypothetical summary of early changes, based on results described in Section 2. The changes occur within approximately the same period as the initial increase in neuronal responsiveness to a visual imprinting stimulus. *Abbreviations*: CaMKII, calcium/calmodulin-dependent protein kinase II; GABA, γ-aminobutyric acid; MARCKS, myristoylated alanine-rich C kinase substrate; PKCγ, protein kinase C γ isoenzyme; P-MARCKS, PKC-phosphorylated MARCKS; PSD, postsynaptic density; P-Ser831-GluA1, GluA1 subunit of AMPA glutamate receptor phosphorylated at Ser-831 by αCaMKII; P-Thr286-CaMKII, Thr286-autophosphorylated αCaMKII. (B) Approximate time-line of changes referred to in (A). The times given are those at which measurements were made; therefore the changes started to occur before these times.

The target of autophosphorylated αCaMKII at the presynaptic site could be synapsin. The synapsins comprise a conserved family of synaptic vesicle-associated proteins, predominantly associated with the reserve pool of synaptic vesicles. Synapsins are phosphorylated by various kinases including CaMKII (Cesca et al., 2010; for review see Greengard et al., 1993). Phosphorylation of synapsin results in the mobilization of synaptic vesicles and increased release of neurotransmitter. During the early stages of imprinting, increased release of GABA, taurine and glutamate is observed (see below). αCaMKII is also present in terminals of GABAergic neurons and is involved in synapsin phosphorylation and presynaptic facilitation of GABA release (Sadanandappa et al., 2013). Therefore activation of αCaMKII could induce increased release of glutamate and GABA from synaptic terminals (see Fig. 3).

### 2.3 Amino acid neurotransmitter release

Calcium-dependent, potassium-stimulated, amino acid neurotransmitter release from the IMM (representing the presumed pool of releasable neurotransmitter) was measured at several times after training with an imprinting stimulus (McCabe et al., 2001, Meredith et al., 2004). Release of aspartate, arginine, citrulline, GABA, glutamate, glycine and taurine were measured. Approximately 6 h after the start of training, only release of GABA and taurine, from slices of the left IMM, were significantly correlated with preference score: these correlations were positive (McCabe et al., 2001). In this connection, it is noteworthy that the learning-related increase in Fos expression in the IMM was restricted to neurons immunopositive for GABA and taurine (Ambalavanar et al., 1999, McCabe and Horn, 1994). Release of these two amino acids was evidently influenced by several processes, not necessarily reflected by the change in Fos expression. Mean release in trained chicks was very similar to, and did not differ significantly from, the mean release for untrained chicks. The evidence suggests that a process predominating in poor/slow learners depresses release of GABA and taurine and that another process predominating in good/fast learners enhances release. In support of these inferences, the variance of release in trained chicks was significantly greater than in untrained chicks. It seems likely that both processes occur subsequent to increased neuronal activity, as indicated by the observed learning-related increase in Fos expression (McCabe and Horn, 1994). The correlations for GABA and taurine release were significant only in the left IMM, whereas the relation between learning and Fos expression is not lateralized (McCabe and Horn, 1994, Suge and McCabe, 2004). Evidence from biochemical (McCabe and Horn, 1994, Suge and McCabe, 2004), ablation (Cipolla-Neto et al., 1982, Horn et al., 1983) and electrophysiological (Brown and Horn, 1994, Horn et al., 2001, Nicol et al., 1995) studies show that both the left and the right IMM contribute to memory for the imprinting stimulus. However, the roles of the two sides are different (cf McCabe, 1991, McCabe, 2013; see also Nicol and Moorman, 2015). The results suggest that the relationship between Fos expression (presumed neuronal activity), and amino acid neurotransmitter release, differs according to the memory process sub-served. In view of the evidence, discussed above, that more than one process affects GABA and taurine release depending on the strength of learning, the lack of significant association with preference score in the right IMM might be due to opposing processes that tend to cancel each other out. A further consideration arises from the finding that the correlation between release of GABA and release of taurine in the left IMM is attributable to the correlation of both of these quantities with preference score (McCabe et al., 2001). When the effect of preference score was removed by partial correlation, release of the two amino acids showed no significant association. Therefore, release of GABA and taurine was correlated as a result of learning, possibly through co-release from the same neurons: when the effect of learning was removed, they were independent. Whether or not these hypotheses turn out to be true, there is evidence that the capacity of synapses in the left IMM to release GABA and taurine is influenced in a learning-related manner for several hours after learning has occurred.

The positive relationship between preference score and GABA/taurine release was confirmed by Meredith et al. (2004), who trained chicks with an imprinting stimulus for just 1 h and measured release 4.5 h, 11 h and 25 h after the start of training. A positive correlation was observed between preference score and release of both GABA and taurine at 4.5 h and 11 h. The learning process of imprinting is therefore associated with increases in releasable pools of GABA and taurine in the left IMM at least 10 h after training has ended. In contrast, no such correlations were observed 25 h after the start of training: the role of GABAergic transmission in memory formation thus changes with time.

Meredith et al. (2004) also found an increase in calcium-dependent, potassium-stimulated release of glutamate in the IMM of trained chicks as compared to untrained ones. In contrast to the results for GABA and taurine, there was no significant effect of time or laterality, and no significant correlation with preference score, suggesting a non-specific effect of exposure to the imprinting stimulus, or possibly a limit to the extent to which glutamate release may be increased. Training with an imprinting stimulus is, nonetheless, associated with a general increase in the releasable pool of glutamate in the IMM, a change which is likely to affect neuronal processing in this region. The possibility exists, of course, that the non-specific increase in glutamate release may act in association with, and perhaps modulates, the changes more specifically related to the behavioural consequences of learning. Taken together with the results from the studies of Fos expression (where the learning-related changes occurred in GABA-immunopositive neurons) and of the GluA1 receptor subunit, it is possible that, during early stages of learning, there is co-modification of glutamatergic and GABAergic transmission.

### 2.4 The role of GABA in memory formation

The function of GABAergic (i.e. inhibitory) neurons in memory formation is unknown, but it is noteworthy that the threshold for associative long-term potentiation of Shaffer collateral-CA1 cell synapses in the mammalian hippocampus is dependent on GABA from feed-forward inhibitory neurons acting on GABA_A_ receptors on the CA1 cells, and on GABA_B_ autoreceptors on terminals of the input neurons. This inhibitory input evidently contributes to the co-operativity that is a definitive property of associative long-term potentiation (Bliss and Collingridge, 1993), and may be important in the synaptic plasticity underlying the memory of imprinting.

A further noteworthy consideration is prompted by the neural network model of imprinting described by Bateson and Horn (1994). An essential feature of that model, in the ‘Recognition’ layer of processing elements responsible for storing a representation of the imprinting stimulus, is the mutual inhibition of processing modules: once a module in this recognition layer is activated by an imprinting stimulus, it inhibits other modules that are less strongly activated by the stimulus, as part of a process that eventually forms a representation of the imprinting stimulus. It would therefore not be surprising if modification of the strengths of GABAergic synapses contributed to formation of memory for the imprinting stimulus.

### 2.5 MARCKS protein

Myristoylated, alanine-rich C-kinase substrate (MARCKS) is a major substrate of protein kinase C (PKC) and plays a key role in G-protein-coupled receptor signalling (see Newton and Johnson, 1998 for review). MARCKS protein is also involved in synaptic vesicle trafficking and neurotransmitter release, through regulation of the actin-containing cytoskeletal structure (for reviews see Larsson, 2006, Sasaki, 2003). MARCKS is present in high concentrations in neurons, both pre- and postsynaptically (Ouimet et al., 1990, Stumpo et al., 1989) where, as in other cells, MARCKS exists in two main forms (Arbuzova et al., 2002, Thelen et al., 1991). In the resting, unphosporylated, state the protein is membrane-bound, where it binds and cross-links actin filaments and/or sequesters phosphatidylinositol-4,5-bisphosphate (PIP2). On phosphorylation by PKC or binding to Ca^2+^/calmodulin, MARCKS translocates to the cytosol, probably releasing actin and PIP2 (see e.g. Arbuzova et al., 2002). In addition, as the cytosolic, phosphorylated form, MARCKS has the capacity to modulate pathways reliant on PKC, Ca^2+^/calmodulin, pre- and post-synaptic vesicle trafficking and nicotinic acetylcholine receptor function (Gay et al., 2008).

Approximately 4 h after the start of training with an imprinting stimulus there is a learning-related increase in the phosphorylation of MARCKS protein by PKC in the left IMM but not in the right. This increase is correlated with the strength of learning (Sheu et al., 1993). No changes were observed in the phosphorylation of a further PKC substrate protein, F1/GAP43, in the IMM. Study of MARCKS and F1/GAP43 mRNA levels after imprinting revealed that the level of MARCKS mRNA was higher in good learner chicks as compared to poor learners. No differences were found in the level of F1/GAP43 (Meberg et al., 1996). Thus, the early stages of learning and memory formation are followed by selective phosphorylation and up-regulation of a PKC substrate – MARCKS protein. This early increase in MARCKS phosphorylation is likely to affect the binding of actin filaments to the plasma membrane (Aderem, 1992, Arbuzova et al., 2002). Were this change to occur in dendritic spines (see Calabrese and Halpain, 2005, Matus, 2005), where actin filaments are abundant (Fifkova and Morales, 1992), the change might permit the early modification in spine morphology that has been observed shortly after imprinting (Bradley et al., 1981, Horn, 1985).

Phosphorylation of MARCKS protein might also be involved in the increased release of GABA, taurine and glutamate taking place at the same time after training (see above and Fig. 3).

Consistent with the above interpretations, many neuronal cell bodies in the IMM are immunoreactive for the PKCγ isoenzyme, and inputs to the IMM are immunoreactive for PKCαβ (van der Zee et al., 1995). Fos labelling, employing the experimental conditions of McCabe and Horn (1994) and Suge and McCabe (2004), was found to be restricted to cells containing PKCγ (Ambalavanar et al., 1993), further characterizing the IMM neurons showing a learning-related increase in Fos expression in the early stages of training as being PKCγ-positive as well as containing GABA, taurine and parvalbumin (Ambalavanar et al., 1999).

### 2.6 Increased inhibition, excitation or both?

The findings concerning molecular changes shortly after training suggest that there is a learning-related increase in inhibition (increase in evoked release of GABA, activation of Fos in GABAergic neurons) as well as an increase in excitatory neurotransmission (GluA1 phosphorylation, increase in evoked release of glutamate). At the same time point, in trained chicks there is an increase in the proportion of neurons responding to the stimulus to which the chick was imprinted (Brown and Horn, 1994, Horn et al., 2001, Nicol et al., 1995, Nicol and Moorman, 2015). It is possible that inhibitory processes are involved in ‘shaping,’ or ‘tuning’ the responses to this stimulus (Horn, 2004).

Consistent with our findings, simultaneous activation of GABAergic and glutamatergic systems is observed in another model of learning and memory. Thus, robust enhancement of excitatory as well as inhibitory synaptic connectivity to and within piriform cortex takes place for several days after learning of an olfactory discrimination task (Cohen et al., 2008, Saar et al., 2012). In this preparation, enhancement of synaptic transmission in cortical neurons is mediated by a robust increase of postsynaptic modulation of AMPA receptor-dependent currents, balanced by enhancement of post-synaptic GABA_A_ receptor-mediated currents (Saar et al., 2012).

### 2.7 Summary of early changes (cf Fig. 3)

Early changes after learning in the IMM are mostly represented by rapid and transitory changes (e.g. phosphorylation of αCaMKII, GluA1, MARCKS; synthesis of proteins with short lifetime such as Fos; evoked neurotransmitter release). Some of the changes discussed are expressed in the left as well in the right IMM, but some of them are restricted to the left hemisphere. At this early time, no quantitative changes were observed in the amount of glutamate receptors (GluA1 subunit, NMDA receptor), GABA receptors (γ4 subunit) or αCaMKII.

## 3 Intermediate changes after imprinting (7–15 h after the start of training)

By approximately 8 h after the start of training with an imprinting stimulus, the proportion of neurons in the IMM specifically responsive to the stimulus’ visual component was found to have fallen significantly, following an approximate doubling of responsiveness after the second, final hour of training (Horn et al., 2001). This intermediate period is characterized by instability of neuronal responsiveness: a large proportion of IMM neurons, which previously responded specifically to the training stimulus, temporarily cease to do so (Jackson et al., 2008). Approximately 25 h after the start of training, strong responsiveness to the visual imprinting stimulus was found to return, provided that uninterrupted sleep was permitted during a period 5–11 h after the start of training (Jackson et al., 2008). During and after this intermediate period, but before the 25 h time point, a number of learning-related biochemical changes appear in the IMM, in parallel with this phase of unstable electrophysiological responsiveness.

### 3.1 NMDA receptors

Imprinting was found by McCabe and Horn (1988) to cause a 59% increase in NMDA-sensitive binding of the excitatory amino acid l-glutamic acid to membranes prepared from the left IMM 11–12 h after the start of training; no such effect was found in the right IMM. The binding assay was conducted at a saturating concentration of the radioligand and consequently the binding of l-[^3^H]glutamate was taken to be a measure of NMDA receptor number. This experiment was prompted by the finding that the mean length of the postsynaptic densities of spine (i.e. putatively excitatory) synapses in the left IMM increased as a result of imprinting training (Bradley et al., 1981, Horn, 1985). In addition to the overall increase in NMDA receptor number, binding in the left IMM was positively correlated with preference score, and partial correlation analysis indicated that the correlation with preference score could not be explained by differences in locomotor activity between chicks. Interpolation of the regression line to a preference score of 50 (i.e. no preference, indicative of no learning) gave an estimated level of binding that was not significantly different from the mean level in untrained chicks. Finally, the variances of the binding in good and poor learners respectively were not significantly different from the variance of untrained chicks, although the *mean* binding differed significantly between good and poor learners. It was concluded that the higher binding in the good learners was a consequence of learning (Horn and Johnson, 1989). In the next series of experiments, changes in NMDA receptor binding was studied in a time-dependent fashion 7, 10 and 12.5 h after the start of training (Horn and Johnson, 1989). Only at the latest time was an effect of training found (McCabe and Horn, 1991). The learning-related increase in inferred NMDA receptor number in the left IMM is therefore delayed in onset. The fact that the change was restricted to the left IMM is consistent with the left-biased laterality found in the effect of training on length of the post-synaptic density of spine synapses (Bradley et al., 1981, Horn, 1985). McCabe and Horn (1988) estimated that, if the extra synaptic membrane contained NMDA receptors (not non-NMDA glutamate receptors) at a constant areal density, a 64% increase in NMDA receptor number would be expected, quite similar to the 59% mean increase actually measured (McCabe and Horn, 1988). The functional significance of the increase in NMDA receptor binding is not known, but there are several interesting possibilities. Increased efficacy of glutamatergic transmission is one; another is an increase in the capacity for NMDA receptor-dependent synaptic plasticity, which might be expected when a representation of the imprinting stimulus is undergoing modification during memory consolidation. A third possibility is that NMDA receptor-containing synapses in the IMM are gated by inputs which modulate the postsynaptic membrane potential, controlling the retrieval of stored information. Postsynaptic NMDA receptors, provided their modulatory sites are occupied appropriately, must both bind glutamate and undergo depolarization in order to pass current (see e.g. Dingledine et al., 1999). If IMM neurons bearing NMDA receptors encode information about the training stimulus, read-out of this information might require (i) re-exposure to the training stimulus and (ii) extra depolarizing input to the neurons (e.g. from a motivational system), which thus gates their activity. Such a mechanism may, for example, determine whether the chick follows a familiar training stimulus. At early stages after training (4 h and 6.5 h after the start of training) no changes were observed either in the left or right IMM (McCabe and Horn, 1991).

### 3.2 γ4 GABA_A_ receptor subunit

In the light of the learning-related changes observed in GABA-immunoreactive neurons in the IMM, it is of interest that there is a delayed reduction in expression of the GABA_A_ receptor γ4-subunit gene, in imprinted chicks as compared to their untrained controls (Harvey et al., 1998). The decrease was observed 14–15 h after the start of training but not at 9–10 h. The level of expression was not related to the strength of imprinting, nor was the effect of training restricted to the IMM: it was also observed in other parts of the mesopallium – the mediorostral nidopallium/mesopallium, a region implicated in auditory imprinting (cf Bredenkotter and Braun, 2000), the entopallium (a visual projection area receiving input from the optic tectum) and the posterior medial nidopallium. The regional distribution of expression of the GABA_A_ receptor γ4-subunit gene in the chick brain is distinctive (Harvey et al., 1998) but the function of this subunit is not known. The results do, however, indicate a re-organisation of inhibitory transmission systems in the IMM and elsewhere, during this intermediate phase following training.

### 3.3 Neurotransmitter release

As already mentioned, potassium-stimulated, calcium-dependent release of GABA and taurine from the slices of the left IMM is positively correlated with preference score 11 h after the start of training, following a similar pattern to that observed at 4.5 h (Meredith et al., 2004) and 6.5 h (McCabe et al., 2001). Imprinting thus evidently gives rise to sustained increases in the releasable pools of GABA and taurine in the left IMM, shortly after the early activation of GABA/parvalbumin/taurine/PKC-immunopositive neurons.

A non-specific increase in release of l-glutamate was observed by Meredith et al. (2004) 4.5 and 11 h after imprinting training. This change was not correlated with preference score, nor did it change significantly with time after training, but persisted for at least a day after training, presumably reflecting a generalized adjustment in excitatory neurotransmitter activity.

### 3.4 Clathrin proteins

Clathrin-coated vesicles are required for a number of receptor-mediated transport events within eukaryotic cells (see Brodsky, 2012 for review). In neurons, clathrin has additionally been implicated in the formation of dense-core secretory vesicles at the trans-Golgi network and in the recycling of synaptic vesicles. Clathrin forms the outer layer of the coated vesicles and surrounds an inner layer of adaptor proteins bound to selected membrane receptors. Clathrin molecules have a triskelion shape; each leg comprises non-covalently-linked clathrin heavy chains, associated with clathrin light chains at the base of each leg.

The maintenance of synaptic transmission requires that vesicles are recycled after releasing neurotransmitter. Imprinting is associated with increased capacity for neurotransmitter release (see above and also this section). Therefore we inquired whether the levels of clathrin heavy and light chains in the IMM change in a learning-related manner 10 and 25 h after the start of a 1-h training period. At the 10 h time point, no significant changes were found either in the left or right IMM or in four control brain regions studied (the left and right sides of the hyperpallium apicale (thalamofugal visual projection area in the forebrain) and left and right PPN) (Solomonia et al., 1997). Effects at 25 h are described below.

### 3.5 Neural cell adhesion molecules

Neural cell adhesion molecules (NCAMs) are members of the immunoglobulin superfamily mediating homo- and heterophilic cell–cell interaction. The NCAM family in adult animals consists of three major members with molecular masses 180, 140 and 120 kDa (reviewed in Ronn et al., 2000). NCAM 180 and NCAM 140 are transmembrane proteins, occur in synaptic membranes and have different distribution between pre- and post-synaptic components (Persohn et al., 1989). NCAMs are involved in synaptic plasticity and can contribute to neuropathological events (Ronn et al., 2000).

We measured the levels of all three major NCAM isoforms 10 h and 25 h after the start of training. As in the case of clathrin proteins, at 10 h no significant effects of training were found on either side of the IMM, the hyperpallium apicale or the PPN (Solomonia et al., 1998); changes at 25 h are described below.

### 3.6 Summary of intermediate changes, lateralization and the effect of sleep

**Fig. 4:**
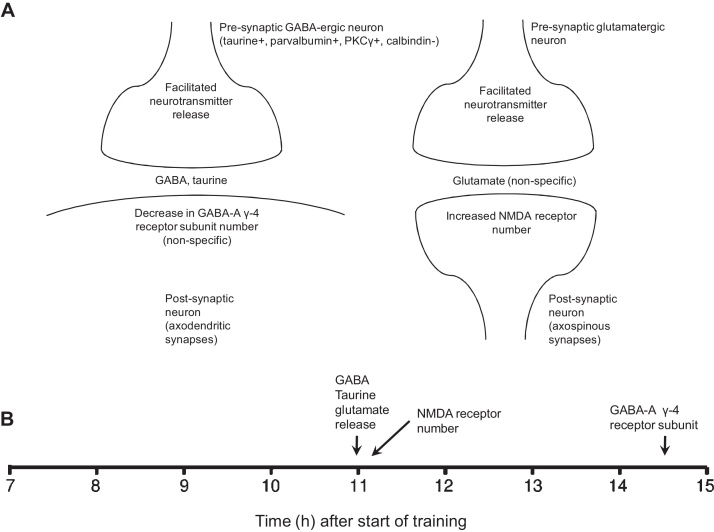
(A) Hypothetical summary of intermediate changes, based on results discussed in Section 3. During this period, the responsiveness of IMM neurons to a visual imprinting stimulus is unstable; uninterrupted sleep over this time establishes stable responsiveness approximately 24 h after the start of training. *Abbreviations*: PSD, post-synaptic density; GABA, γ-aminobutyric acid; NMDA-R, N-methyl-d-aspartate receptor. (B) Approximate time-line of changes referred to in (A). The times given are those at which measurements were made; therefore the changes started to occur before these times.

See Fig. 4 for a summary of intermediate changes. Within a few hours of the start of training, lateralization is apparent in some learning-related changes. In particular, the increases in axospinous PSD length (Bradley et al., 1981, Horn et al., 1985) and NMDA receptor number (McCabe and Horn, 1988) are restricted to the left IMM. These results raise the question of whether the asymmetries depend on previous sensory experience, since illumination of the egg between the 19th and 21st days of incubation stimulates the right eye more than the left owing to the position of the embryo's head relative to the rest of its body. The asymmetric stimulation results in anatomical and functional asymmetries in the thalamofugal visual pathway (see Rogers, 2012 for review). However, the eggs used for the imprinting experiments described above were incubated in darkness and therefore the ensuing hemispheric asymmetries cannot be attributed to differential stimulation of the right and left eyes.

The question also arises as to why neuronal responsiveness to the imprinting stimulus in the IMM is highly variable shortly after training, and becomes stable after uninterrupted sleep (Jackson et al., 2008). The high variability suggests instability of synaptic connections. The neural representation of the imprinting stimulus may accordingly be particularly amenable to modification during this variable phase, allowing certain components of the memory trace to be strengthened and others to weaken or disappear. For example, connections that are most frequently activated, or which encode particularly salient stimulus features, might be selectively strengthened. A mechanism of this sort can improve the efficiency of certain neural networks (in particular Hebbian networks) by protecting them against saturation and erroneous recall of input activity patterns that are correlated with one another (cf Gardner-Medwin, 1989). Thus, a short-term memory working in conjunction with a long-term memory, with the flow of information between the two requiring sleep, may confer both selectivity and economy of storage on a memory system. It is of interest that the postsynaptic density of axospinous synapses and the numerical density of NMDA receptors in the left IMM increase at approximately the time that sleep is effective in stabilization (Fig. 3, Fig. 4). These lateralized learning-related changes in IMM excitatory synapses, suggesting enhanced synaptic plasticity, may reflect conditions necessary for the late changes in proteins observed in the IMM and discussed below.

## 4 Late changes (>15 h after the start of training)

This period is characterized by the highest level of responsiveness of neurons to visual characteristics of the imprinting stimulus. The proportion of neurons in the IMM thus tuned to the imprinting stimulus is approximately three times that measured just before training and is also significantly higher than that at the 8 h time point (Horn et al., 2001). A number of learning-related molecular changes appear at this time of high, stable responsiveness to the imprinting stimulus.

### 4.1 Neuronal cell adhesion molecules (NCAMs)

The amounts of all three major NCAM isoforms (molecular weights 180, 140 and 120 kDa) were found to be increased in a learning-related way in the left IMM 25 h after the start of training. No significant changes were observed in the right IMM or in four control regions – the left and right hyperpallium apicale and the left and right PPN (Solomonia et al., 1998). The three NCAM isoforms have different cellular and subcellular localizations and the increase in all three isoforms suggests several types of learning-related change.

Oligodendrocytes express mainly NCAM 120 (Bhat and Silberberg, 1988). The increased expression of NCAM 120 in the left IMM at 25 h could reflect a change in the rate or pattern of myelination. In post-synaptic densities (PSDs) from the IMM, NCAM 180 is enriched relative to other isoforms (Solomonia et al., 1998). At least part of the learning-related increase in the amount of NCAM 180 might occur in the PSD. NCAM 180 could then interact presynaptically with NCAM 140 by means of heterophilic binding and also with cytoskeleton proteins (Pollerberg et al., 1987). One might therefore speculate that learning-related increases in the amounts of NCAMs at 25 h (but not at 10 h) may serve to stabilize, through direct adhesive and intracellular interactions, certain synapses that are modified through learning. This stabilization might even terminate the period of plasticity at these synapses.

### 4.2 Clathrin proteins and neurotransmitter release

At 25 h after the onset of training, the amount of clathrin heavy chain is increased in a learning-related manner in the left IMM, but not in the right or in four control brain regions studied (Solomonia et al., 1997). In contrast, no significant changes were observed for the light chain. Functional triskelions are assembled from the non-covalent interaction of both heavy and light chains. Studies in vitro suggest that clathrin heavy chain is probably the limiting component in triskelion biosynthesis (Brodsky, 1985). Thus, the learning-related increase in amount of clathrin heavy chain may reflect control of triskelion synthesis. However, it should be born in mind that the light chains are synthesized in excess (Brodsky, 1985); therefore any changes in amount of light chain may be difficult to detect against the high basal level. The major function of neuronal clathrin is to recycle synaptic vesicle proteins following exocytosis. Potassium-stimulated, calcium-dependent release of GABA and taurine, whilst significantly correlated with strength of learning up to 11 h after the start of training, ceased to be so at 25 h. In contrast, release of glutamate in trained chicks persisted up to this time (McCabe et al., 2001, Meredith et al., 2004). At the early and intermediate stages after training, the amount of clathrin may not be rate-limiting for vesicle recycling. However, if the turnover of synaptic vesicles is elevated over a long period, there may be a need for a corresponding increase in levels of clathrin such as that found in our study (Solomonia et al., 1997). This increase may reflect a long-term change in the dynamics of transmitter processing associated with an up-regulation of neurotransmitter release at selected synapses of IMM. The data also suggest an important role for presynaptic events in the IMM during memory formation (Solomonia et al., 1997, Solomonia et al., 2005, Solomonia et al., 2013).

### 4.3 Gene expression and net protein synthesis

Analysis of time-dependent, learning-related changes after training raises the possibility of selective, time-dependent changes in gene expression patterns. We used suppressive subtractive hybridization (SSH) to identify genes, the mRNAs of which are up- or down-regulated in the IMM 25 h after the start of training (Solomonia et al., 2003, Solomonia et al., 2011). Experiments were conducted using IMM samples pooled from two groups of chicks. One pooled sample was from a group that had developed a strong preference for an imprinting stimulus (good learners) and the other pooled sample was from a group that did not develop a preference (poor learners). We identified more than 40 differentially expressed genes, but in this context up- or down-regulation of a particular gene alone does not necessarily imply that the changes were related to learning. This is because only two IMM samples were compared. To validate the results obtained we enquired whether the levels of protein products identified by the differentially expressed candidate genes were related to the strength of learning (Solomonia et al., 2003, Solomonia et al., 2011).

#### 4.3.1 Amyloid precursor protein (APP)

The level of APP in the left IMM of trained chicks was up-regulated by training, was strongly correlated with preference score and satisfied the criteria for a learning-related change (Solomonia et al., 2003). In the right IMM the amount of APP in the chicks with medium and high preference scores was significantly higher than that of untrained chicks, but did not correlate with preference score. No significant changes were found in the PPN (Solomonia et al., 2003).

APP is a ubiquitously-expressed type I transmembrane protein that undergoes regulated proteolytic processing by various secretase enzymes to yield proteolytic fragments. The processing of APP via the amyloidogenic pathway results in the generation of amyloid-peptide, which is the main constituent of extracellular amyloid plaques – the hallmark of Alzheimer's disease. The function of APP remains incompletely understood (Wolf and Guenette, 2007). Many different functions have been assigned to this protein or its various fragments, including cell-surface receptor, trafficking, regulation of transcription, adhesion, neuroprotection (for soluble APP) and others (see e.g. Wolf and Guenette, 2007 for review). Below we discuss some of the features of this protein which are relevant to the changes occurring after imprinting.

APP associates with NR2A and NR2B subunits of NMDA receptors, resulting in the enhancement of the delivery of these receptors to the cell surface (Cousin et al., 2009). It has been shown further that APP is a constituent of the PSD and co-immunoprecipitates with subunits of NMDA receptors (Hoe et al., 2009). Taken together, our results provide evidence for an increase in PSD size and for an increase in certain protein components of the PSD such as NCAM 180 (Horn, 1985, Solomonia et al., 1998) 25 h after training onset. The change in APP might contribute to this change in the PSD. In this connection, and in consideration of the finding that NMDA receptor number in the left IMM is increased as a result of training, it is noteworthy that NMDA receptors have been implicated in imprinting in the chick by others (Nakamori et al., 2010, Nakamori et al., 2011, Nakamori et al., 2013).

APP may also have a presynaptic function. The protein accumulates in the fraction containing docked synaptic vesicles, but is not associated with free synaptic vesicles. It has been concluded that APP is a constituent of the presynaptic active zone (Labek et al., 2013). The coincidence of learning-related increases of APP and clathrin heavy chain (Solomonia et al., 1997) might reflect a coordinated increase in the components of the machinery involved in synaptic vesicle docking and release.

#### 4.3.2 MARCKS protein

According to the study employing SSH, the level of mRNA coding for MARCKS protein was elevated in the left IMM of strongly imprinted chicks as compared to poorly imprinted chicks 25 h after the start of training (Solomonia et al., 2003). During the early phase after learning, MARCKS protein phosphorylation by PKC and the level of MARCKS mRNA was found to increase with learning (Meberg et al., 1996, Sheu et al., 1993). Therefore, one of the candidate genes which was investigated at 25 h was that encoding MARCKS. The amount of MARCKS protein in nuclear-free homogenates of the left IMM 25 h after the start of training was found to increase significantly with preference score. No learning-related changes were observed in the right IMM or left or right PPN (Solomonia et al., 2003). These experiments were carried out on a homogenate fraction containing both cytoplasmic and membrane pools of MARCKS. The differences in sub-cellular localization reflect different functional states of MARCKS protein (phosphorylation, calmodulin binding). Therefore, in the next series of experiments we inquired whether the learning-related increase in amount of MARCKS observed in the IMM 25 h after the start of training is restricted to the PKC-phosphorylated form, to the membrane-bound form, or whether both of these forms of the protein increase with learning. The learning-related increase was observed only for the membrane-bound, non-phosphorylated form of MARCKS and not for the cytoplasmic, phosphorylated form (Solomonia et al., 2008). These results, together with previous ones (Sheu et al., 1993) imply that the early changes in MARCKS acting through the PKC-phosphorylated form may be involved in early memory formation. The later increase in only the non-phosphorylated, membrane-bound form may stabilize early changes by strengthening actin binding to the membranes in dendritic spines (Solomonia et al., 2008).

#### 4.3.3 Fodrin

In the SSH studies the mRNA level for α-fodrin was down-regulated. α-Fodrin (also known as brain spectrin), is a membrane-cytoskeletal linker protein (Malchiodi-Albedi et al., 1993) and has been implicated in synaptic plasticity (Lynch and Baudry, 1984, Lynch et al., 2007). The study of the changes in the amount of protein showed that the level of α-fodrin in the left IMM was negatively correlated with preference score but that the mean level of α-fodrin was not significantly different from the mean untrained value.

#### 4.3.4 Mitochondrial proteins

Amongst differentially expressed transcripts in the SSH study there were a number from the mitochondrial genome, namely t-RNAs, subunits of ATPase and subunits I and II of cytochrome c oxidase (COI and COII). The enzyme cytochrome c oxidase is a mitochondrial protein complex that plays a crucial role in oxidative metabolism; indeed, mitochondria play a role in many aspects of neuronal function including, it has been suggested, synaptic changes that underlie learning and memory (Li et al., 2004, Mattson et al., 2008). We inquired whether the levels of COI and COII correlated with the strength of learning 25 h after the start of training. Learning-related increases in the amounts of COI and COII were found only in the left IMM and not in the right IMM or the left or right PPN (Solomonia et al., 2011).

**Fig. 5:**
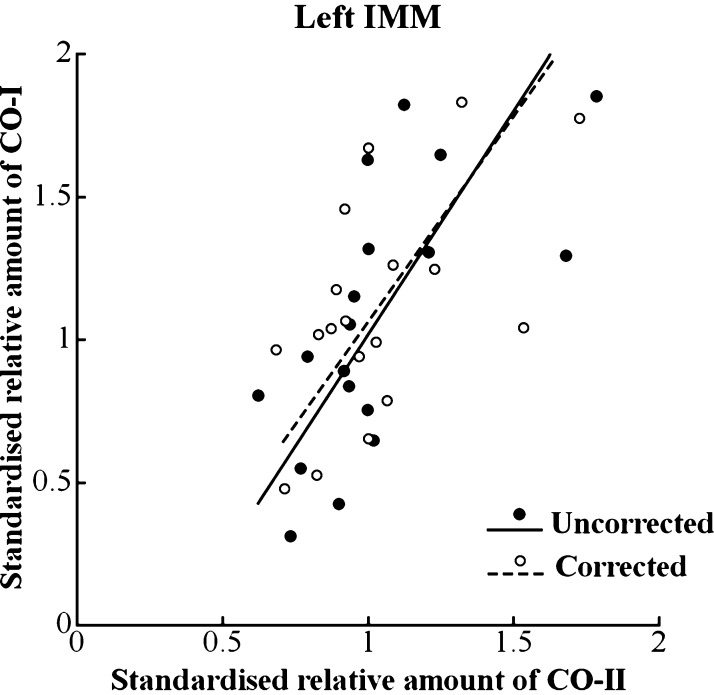
Left IMM. Amount of COI plotted against amount of COII after training for 1 h. Closed symbols, uncorrected for preference score; open symbols, corrected for preference score by partial correlation. Standard major axes have been fitted respectively to the uncorrected data (solid line) and corrected data (broken line). Uncorrected data: correlation coefficient *r*_16_ = 0.67, *p* = 0.0024; slope of fitted line 1.57 ± 0.29 SE Corrected data: partial correlation coefficient *r*_*xy*·*z*;15_ = 0.58, *P* = 0.015; slope of fitted line, 1.44 ± 0.30 SE. The correlation coefficient (*r*) and slope of the fitted line for trained and untrained chicks combined (*r*_23_ = 0.67, *P* < 0.001; slope = 1.37 ± 0.21) were not significantly different from the corresponding values (whether corrected or uncorrected) for the trained chicks alone. There was significantly more COI and COII in trained than in untrained chicks (COI, *F*_1,28_ = 5.78, *P* = 0.023; COII, *F*_1,23_ = 10.85, *P* = 0.0032).

Reproduced with permission from Solomonia et al. (2011).

It was found that there was a strong correlation between the amounts of COI and COII in the left IMM of trained chicks, but not in the other brain regions studied (Fig. 5). Such a correlation would be expected when the amounts of COI and COII respectively increase with the strength of learning. However, the correlation between the two proteins remained significant even after correcting for preference score by partial correlation analysis. This finding suggests that learning did not influence the relationship between the two proteins. Indeed, the correlation between amounts of the two proteins was virtually unaffected by adding data from untrained chicks to those of trained chicks. Instead, it was concluded that the correlation between amounts of the two proteins may be a characteristic feature of the left IMM (Solomonia et al., 2011).

There are at least two reasons why the absence of significant correlation between the COI and COII in three other brain regions was unexpected: (i) the genes coding for COI and COII are located adjacent to each other and are transcribed together (Kelly and Scarpulla, 2004); (ii) subunits COI and COII, encoded in mitochondrial DNA, contribute in equal numbers to the assembling enzyme complex (Khalimonchuk and Rodel, 2005). Hence the disassociation between COI and COII in control brain regions or in the right IMM is unlikely to be attributable to a de-correlation in the *expression* of the two subunits on mRNA level or composition of already assembled cytochrome c oxidase complex. It was suggested that the reason for this disassociation lies in the process of assembly of the enzyme complex – cytochrome c oxidase is assembled from subunits, some of which are encoded by mitochondrial and some by nuclear DNA. Many factors are involved in the assembly of the subunits and in the insertion of cofactors and metal ions into the maturing enzyme complex (Fontanesi et al., 2006, Mick et al., 2011). A less well-understood group of proteins is involved in stabilizing immature CO pre-complexes (Khalimonchuk and Rodel, 2005). It is possible that the left IMM contains all the factors necessary for assembling the enzyme but that they are absent or less abundant in the other regions studied, so that in the latter, incorporation of the subunits into the assembling complex is less coordinated. As a result, one or other or both of the unassembled subunits degrade (Fontanesi et al., 2006). A difference in the rates of degradation of the two subunits would account for the observed disassociation between amounts of COI and COII. The positive correlation between the amounts of COI and COII in the left IMM and the absence of a significant correlation in the other brain regions raises the possibility that the left IMM contains a greater proportion of combined to uncombined subunits and a greater amount of the assembled enzyme complex than the other regions. This possibility is of interest in the light of the specific role the region plays in learning and memory. The domestic chick being precocial, learns rapidly shortly after hatching. The neural processes that are engaged in the formation of the memory trace place a demand on oxidative metabolism and hence on CO. It is possible that the molecular mechanisms involved in the coordinated assembly of this enzyme complex are precociously developed in the left IMM, enabling this region in particular to function as a memory store within hours after the chick has hatched (Solomonia et al., 2011).

#### 4.3.5 Proteomic studies

Our data indicate that learning gives rise to changes in membrane-mitochondrial protein composition after imprinting in the left IMM. For a more wide-scale study of these changes we have applied two-dimensional gel electrophoresis analysis to synaptic plasma membrane-mitochondrial fractions from the left IMM 25 h after the start of training. Proteomes of the left IMMs from good learners, poor learners and untrained chicks were compared. Significantly differentially expressed bands were further analyzed by mass spectrometry in order to identify them. Several proteins were identified and the involvement of many of them in the imprinting process was validated by correlation analysis (unpublished data). We will discuss only one of those proteins, namely cognin. This protein has been identified as cell adhesion molecule in chick retina, with a high homology to protein disulphide isomerase (PDI) (Krishna Roa and Hausman, 1993). Further, it has been shown that mRNAs for chicken PDI and cognin are identical (Pariser et al., 2000). PDI/cognin resides in the endoplasmic reticulum and it is suggested that it escapes to the cell surface after cleavage from its endoplasmic reticulum retention signal, a c-terminal motif by which PDI/cognin is recognized and bound to a receptor that causes the molecule to remain in the endoplasmic reticulum (Capitani and Sallese, 2009, Pariser et al., 2000). Cognin possesses protein disulfide isomerase, prolyl-4-hydroxylase and thyroid hormone-binding activities and uses disulfide exchange activity to modulate cell adhesion (Pariser et al., 2000).

We have generated specific antibodies against a peptide from the C-terminus of chicken cognin and measured the amount of cognin in the cytoplasmic and P2 membrane fractions of chick brain 25 h after the start of imprinting training. The amount of membrane-bound, but not cytoplasmic, cognin increased significantly with the strength of learning in the left and right IMM, but not in the PPN (unpublished data). These data and our results with NCAM suggest enhanced cell adhesion at late stages of memory formation.

**Fig. 6:**
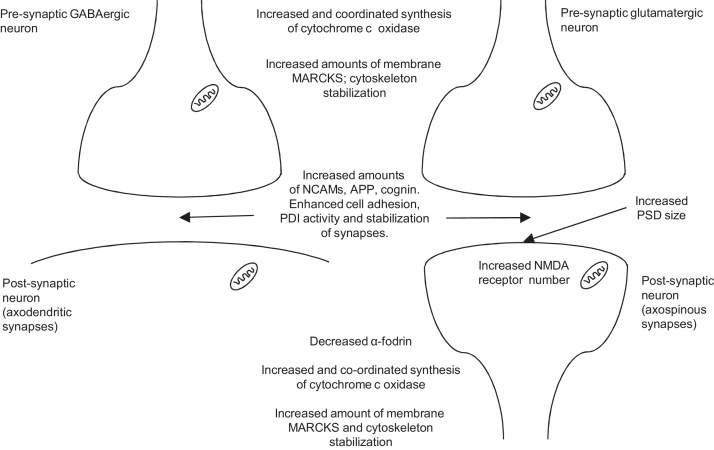
Hypothetical summary of late changes, based on results discussed in Section 4. *Abbreviations*: APP, amyloid precursor protein; GABA, γ-aminobutyric acid; NCAM, neuronal cell adhesion molecule; MARCKS, myristoylated alanine-rich C kinase substrate; NMDA, N-methyl-d-aspartate; PDI, protein disulphide isomerase; PSD, postsynaptic density. All measurements made ∼25 h after the start of training.

See Fig. 6 for a summary of late changes.

## 5 Conclusion

A series of molecular changes after learning has been described in the IMM, previously identified as a memory system. Electrophysiological results show that memory processing in the IMM has a distinct temporal profile, characterized by an initial increase in neuronal responsiveness to the imprinting stimulus, which wanes temporarily after training for 2 h. The decrease in responsiveness coincides with a decrease in response reliability, during a period when uninterrupted sleep appears necessary, for both reliable neuronal responsiveness and behavioural expression of imprinting the following day. Ultimately, provided that uninterrupted sleep has been permitted in a period 5–11 h after training onset, a strong enhancement of neuronal responsiveness is observed. This temporal profile can conveniently be divided into early, intermediate and late phases, each coinciding with particular learning-related biochemical changes:

• *The early phase* is characterized by increased expression of Fos in an identifiable subset of GABA-, taurine-, parvalbumin and PKC-γ-immunopositive neurons, increased phosphorylation of presumed synaptic proteins and enlarged releasable neurotransmitter pools of GABA, taurine and glutamate.
• *The intermediate phase* is also associated with enhanced releasable pools of these neurotransmitters, but also exhibits an increase in NMDA receptor number that was not present in the early phase.
• *Between 15 and 25* *h after training onset (late phase)*, learning-related changes in the amount of protein occur, leading to stable synaptic morphological changes and coinciding with enhancement and greater reliability of neuronal responsiveness (contingent on sufficient sleep).

Recent methodological advances, as well as providing opportunities for identifying the neural signature of an imprinting stimulus, also present challenges. The advances are yielding more detailed electrophysiological and biochemical information. The challenges continue to lie in the task of identifying changes, within the temporal profile described here, that are critical for memory. Equally important is the requirement to identify processes, revealed by separate analytical techniques, which are complementary manifestations of these same critical stages.
